# Experiences of Participants with Spinal Cord Injury at an Active Rehabilitation Camp

**DOI:** 10.3390/jfmk9010007

**Published:** 2023-12-25

**Authors:** Terese Wilhelmsen, Anne Marie Lannem, Marit Sørensen, Marika Augutis, Henrik Gustafsson

**Affiliations:** 1 Department of Educational Sciences, University of South-Eastern Norway, 3045 Drammen, Norway; terese.wilhelmsen@usn.no; 2 Research Department, Sunnaas Rehabilitation Hospital, 1450 Nesoddtangen, Norway; lannem@online.no; 3 Department of Sport and Social Sciences, Norwegian University of Sport Sciences, 0806 Oslo, Norway; henrik.gustafsson@kau.se; 4 Department of Neurobiology, Care Sciences and Society, Division of Neurogeriatrics, Karolinska Institutet, 141-83 Stockholm, Sweden; marika.augutis@ki.se; 5 Department of Educational Sciences, Karlstad University, 651 88 Karlstad, Sweden

**Keywords:** active rehabilitation, fitness, fuzzy qualitative comparative analysis, physical activity camps, psychological benefits, social benefits, spinal cord injury

## Abstract

This study explored the physical, social, and psychological benefits of an active rehabilitation (AR) camp as experienced by participants with spinal cord injury (SCI), and perceived fitness and mastery of being physically active six months after the camp. The study used a mixed-method design with pre- (*n* = 23), post- (*n* = 23), and follow-up questionnaires (*n* = 18) and individual interviews (*n* = 8). Fuzzy qualitative comparative analysis (fsQCA) was used to analyze the quantitative data and qualitative content analysis was used to analyze the qualitative data. Results showed that benefits were mainly experienced in the social and psychological domains. As for the physical domain, younger and more recently injured persons with tetraplegia reported more benefits. Six months after the camp, being in the preparation stage of change and being somewhat physically active were necessary and sufficient conditions for experiencing mastery of physical activity regardless of injury type, but only persons with paraplegia experienced fitness benefits. Qualitative data shed further light on the perceived benefits of the camp. The knowledge gained from this study might help practitioners to tailor interventions to individual needs and researchers to ask questions that take into consideration the complexity of active rehabilitation and changes in physical activity behavior for people with SCI.

## 1. Introduction

Physical activity promotes a healthier life, psychological well-being, and fewer com-plications for people living with spinal cord injuries (SCIs) [1,2,3]. Even so, the level of physical activity is often low in this population [4,5]. Research has identified important barriers and facilitators to physical activity experienced by persons living with SCIs [6,7]. Examples of barriers are problems with accessibility, physical health, self-care, and mental health, the latter being the most important shortly after discharge [6,8,9,10]. Important facilitators identified are preparation for daily physical and social activities during rehabilitation and stimulation to be more physically active [10].

To change sedentary lifestyle and exercise habits, different motivation strategies can be used. One possibility is active rehabilitation (AR) camps [11]. AR camps are camps arranged outside of specialist health care [11]. The aim is to support persons with SCIs to reach their full potential for activity and participation. A central psychosocial factor in AR camps is the use of peer mentors [11]. Research on peer mentors for people with SCIs has so far mostly been reported from community-based programs [12,13].

In this study, we explore the psychological, physical, and social benefits of a Nordic AR camp as experienced by participants with SCI, as well as the participants’ perceived fitness and exercise mastery six months after the camp. The research project was motivated by the limited research-based knowledge regarding AR [11,14].

Most of the research exploring physical activity rehabilitation among people with SCI has been framed within a predominantly medical model of understanding disability. Physiological benefits and effects of specific training have been the focus [15,16]. However, objective measures of function and impairment alone are not adequate to explain the variation in physical activity among people with SCI after AR. Some psychological variables, such as quality of life (QoL), resilience, self-efficacy, and life satisfaction, are sometimes included, but seldom with a theoretical rationale or explanation [11,17]. The theories behind these concepts may help in understanding how to best maximize the benefits of AR camps [18]. Therefore, in this study, we applied a biopsychosocial model and used a theoretical framework to study the benefits of an AR camp as experienced by participants with SCI.

### 1.1. Theoretical Framework

One framework for studying readiness to engage in physical activity is the stages of change framework, also called the transtheoretical theory [19]. Prochaska and DiClemente experienced that regardless of the type of behavior or what theoretical base was used for helping behavior change, people seemed to move through certain stages in changing their behavior. Originally developed for addictive habits, the framework was applied to adoption and maintenance of physical activity by Marcus and Simkin [20]. The stages are precontemplation, contemplation, preparation, action, and maintenance, each stage describing different mindsets as to readiness for engaging in physical activity. Precontemplation is the least ready state, whereas those at the maintenance stage are physically active regularly. Each stage demands different motivational strategies and approaches.

One psychological variable relevant to all stages is self-efficacy, or the person’s belief in their capacity to execute behaviors necessary reach specific goals [21,22]. Developed mainly by Albert Bandura, the theory of self-efficacy is named social cognitive theory [21], and posits that personal, behavioral, and environmental factors influence people’s behavior. Personal factors incorporate perceived self-efficacy and outcome expectations related to the specific behavior. 

The use of peer mentors is in line with social cognitive theory [21]. According to this theory, role learning and vicarious experience are among the important ways of increasing self-efficacy [21]. This can increase the likelihood of moving to a higher stage of readiness for being physically active [23]. Research has demonstrated a nearly linear relationship between the stages of change and physical activity self-efficacy, with the lowest self-efficacy in the precontemplation stage, and the highest in the maintenance stage [24]. A study by Ginis et al. [25] found that variables consistent with social cognitive theory accounted for 39.4% of the variance in leisure time physical activity among people with SCIs. Other SCI-related studies have indicated that self-efficacy, pain intensity, perceived social support, and perceived health account for variations in QoL among people with SCI [26,27]. 

A recent study investigated the role of peer mentorship on QoL and participation for individuals with SCIs and tested if another psychological model, self-determination theory [28], could explain the role of SCI peer mentorship for those outcomes [29]. They found that satisfaction of the need for competence and relatedness mediated the relationship between peer mentorship and outcomes as to QoL and some participation variables. Years since injury also modified the same relationship. This demonstrates how a sound theoretical rationale gives information that may improve practice. 

A scoping review on the topic of peer mentoring concluded that the number of studies were small and recommended further research to support peer mentoring as a viable resource in SCI rehabilitation [30].

### 1.2. Research Questions

As an answer to that call, and to deepen our understanding of the psychosocial processes involved in AR camps, the purpose of this study was not to evaluate whether the pronounced aims of the camps were achieved, but to investigate the psychological, physical, and social benefits of a physical AR camp as experienced by participants with SCIs, as well as the participants’ perceived fitness and exercise mastery six months after the camp. Three research questions guided this study:

1. What are the necessary and/or sufficient conditions for participants with SCIs to experience psychological, physical, and social benefits of a physical activity camp?
2. What characterizes the camp experiences shared by participants?
3. What are the necessary and/or sufficient conditions for perceived fitness and exercise mastery six months after the camp?

## 2. Materials and Methods

### 2.1. Design and Procedures

This study used a natural non-experimental mixed-method design with pre-, post-, and follow-up questionnaires and semi-structured interviews [31]. Participants answered a survey prior to the camp, directly after the camp, and a follow-up survey six months after the camp. In addition, eight participants took part in a semi-structured individual interview during the camp. The duration of the interviews lasted between 14 and 25 min and was conducted by two of the authors, both experienced in qualitative interviewing and with long experience of SCI rehabilitation and AR. They were not involved in organizing the camp and had not been involved in the previous care of the participants in between or after the activities.

### 2.2. Setting

The research took place during a six-day Nordic summer AR camp for people with SCIs (Camp Spinal). Due to the low incidence of people with SCIs, the camp was a Nordic collaborative initiative between Norway and Sweden. Norwegians and Swedes can understand each other’s languages, so no language adaptions are needed. The aim of Camp Spinal was to improve independence by focusing on capabilities and skill achievements, and to promote self-efficacy and physical activity participation with peer learning strategies. Peer leaders and instructors with SCIs acted as role models. During the camp, participants were introduced to a range of physical activities and took part in social events. Examples of physical activities introduced were orienteering, swimming, basketball, rowing, strength training, and wheelchair technique. In addition, social events such as dinners and dialog groups on social life issues were introduced in the evenings for the participants. Most activities were arranged with the intention to create heterogenous groups in terms of age, type of injury, and time from injury. The camp aimed to provide sufficient time for restitution between activities. All participants were accommodated at the same hotel where they shared rooms.

### 2.3. Participants

Altogether, 30 participants with SCIs took part in the same AR camp. Participation was voluntary and on personal initiative. Twenty-five of the participants responded to the survey the first day at the camp, eight participants took part in individual interviews during the camp, and eighteen participants responded to a six-month follow-up survey. Of the 25 pre-camp respondents, 1 was excluded from the data due to being aged below 15, and 1 was excluded due to inconsistences in the responses. Relevant participant characteristics are summarized in groups in Table 1 in the results section to protect the participants’ anonymity.

### 2.4. Measures in the Questionnaires

To measure psychological, physical, and social benefits, we used three items developed to evaluate the experienced benefits of taking part in the camp. Psychological benefits were measured by the item: “You have been very active this week. Has this made you mentally stronger?” Physical benefits were measured by the item: “You have been very active this week. Has this made you physically stronger?” It should be noted that during the camp the participants performed physical activities for around six hours per day, so stating that they had been very active that week was based on this fact and should not prime the responses in any direction. Social benefits were measured by the item: “Has the social community meant something for you this week?” All items were responded to by indicating on a visual analogue scale ranging from 0–10 (0 = no, not at all; 10 = yes, to a very large degree). Visual analogue scales are widely used as measures of several psychological variables and have demonstrated acceptable psychometric properties [32].

Perceived exercise mastery is defined as perceived competence in the execution of physical exercise [33]. To measure perceived exercise mastery and perceived fitness, we used the five-item mastery scale and the three-item perceived fitness scale in the Self-Perception in Exercise Questionnaire (SPEQ) [33]. The reliability and validity of the SPEQ have been documented in a Norwegian able-bodied population and in a SCI population [33,34]. An item example from the perceived exercise mastery scale is “Doing physical activities is important to me, among other things, because then I feel that I really master something”. An item example from the perceived fitness scale is “I am overall in a bad shape”. Each item was scored on a 4-point scale (1 = completely disagree; 4 = completely agree), and a mean score for each scale was computed.

The stages of change applied to physical activity were inspired by Marcus and Simkin [20]. The instructions were: ‘‘Physical activity. Choose only one alternative. Regularly physically active means minimum of three 20 min session physical exercise a week”. The response options for the scale were: (1) I am not regularly physically active and I have no plans to change physical activity behavior within the next six months (precontemplation), (2) I am not regularly physically active but I plan to become physically active within the next six months (contemplation), (3) I am not physically active, but I plan to become physically active in the near future, within one month (preparation), (4) I am regularly physically active, and have been so six months (action), and (5) I am regularly physically active, and I have been so for more than six months (maintenance). Many studies have demonstrated satisfactory psychometric properties for this measure of stages of change. Marcus and Simkin [35] demonstrated concurrent validity, and Sarkin et al. [36] reported concurrent and construct validity for the measure among an overweight population.

Physical activity level was measured with the leisure time physical activity (LTPA) one-item categorical scale in which participants are asked to rate their weekly average LTPA: (1) reading, watching TV or other sedentary activities; (2) walking, cycling, using a wheelchair or moving about in some other way at least 4 h per week—including commuting to work and Sunday walks; (3) taking part in physical exercise or sports, heavy gardening work, etc.—at least 4 h a week; and (4) exercising hard or taking part in competitive sports regularly several days a week. The scale is widely used in Norwegian health surveys and SCI research [37,38].

Participants were also asked to describe the neurological level and degree of SCI (i.e., paraplegia, tetraplegia, complete or incomplete) and the year of injury in the pre-camp survey.

### 2.5. Qualitative Data

The qualitative data consisted of responses from five open questions from the surveys and interview data. Questions from the pre-camp survey were related to expectations and aims towards the camp, and questions from the post-camp survey were related to the meaning the participants ascribed to the camp (i.e., “What has the camp meant for you?”), as well as positive and negative experiences with the camp. In the semi-structured interviews, five aspects were explored: (1) reason for choosing the camp; (2) expectations and aims before the camp; (3) experiences of participating in activities, (4) experiences of social interaction with other participants and leaders, and (5) post-camp expectations.

### 2.6. Data Analysis

#### 2.6.1. Fuzzy Qualitative Comparative Analysis of the Survey Data

To analyze the survey data, we used fuzzy qualitative comparative analysis (fsQCA). FsQCA is an analytical approach used to explore the causal complexity in a data set by examining cases that share combinations of conditions to see if they also share the same outcome, and to interpret relations between the conditions and the outcome in terms of necessity and/or sufficiency [39]. FsQCA is a case-based approach using both qualitative and quantitative data derived from cases to identify what conditions, or combination of conditions, are necessary and/or sufficient for an outcome. A strength of the case-based nature of fsQCA in comparison with variable-based traditional regression analysis is that it allows for rigorous analysis of the causal complexity of behavior change in studies limited by a small number of cases, which is often the case with SCI research [40]. FsQCA is still unknown for many, so in the following section we briefly introduce the underlying principles and components of the approach.

FsQCA is based on three underlying principles. First, equifinality—more than one pathway to an outcome is possible. Second, conjunctional causation—a condition may not affect the outcome alone but may do so in combination with another condition. Third, asymmetrical causation—both the presence and absence of a condition may lead to the outcome depending on the presence or absence of other conditions. In short, fsQCA allows us to explore alternative pathways to experiencing physical, psychological, and social benefits of the AR camp, while considering the effects of different configurations of the presence or absence of specific conditions.

A condition is necessary if, whenever we see the outcome, then we also see the condition. However, necessity does not equivalent to sufficiency. A condition is sufficient if whenever we see the condition then we also see the outcome. As an example, all people with SCIs have a neurological injury. Thus, a neurological injury is a necessary condition for SCI but not a sufficient condition, as many people with a neurological injury do not have an SCI. To test for necessity and sufficiency, fsQCA uses formal logic and Boolean algebra.

In contrast to the conventional crisp QCA, in which a case is dichotomously coded as either “in” or “out” of a set, fuzzy sets permit membership in the interval between 0 and 1. Thus, fuzzy membership scores imply the degree to which different cases belong to a condition (including full membership, the point of crossover, and full non-membership; [39]). Membership scores in the range 0.5–1 represent cases that are more “in” than “out” of a given condition. Scores in the range 0–0.5 represent cases more “out” than “in” of a condition, and the score 0.5 represents the point of maximum ambiguity neither “in” nor “out” of the condition.

The measures of fit reported on are consistency (con), indicating the degree to which cases with the outcome also exhibit the conditions. In the analysis, we use the minimum consistency score of 0.90. Raw coverage (cov.r) measures the degree to which the conditions in the solution formula explain all cases with the outcome. Unique coverage (cov.u) measures the partitioning coverage of each configuration in the formula. The proportional reduction in consistency (PRI) measures the reduction inconsistency if one configuration is left out of the model.

We used R version 4.1.3 (R Core Team, Vienna, Austria) as platforms for the analyses. To perform the fsQCA analyses, we used the R package “QCA” [41].

#### 2.6.2. Content Analysis of the Qualitative Data

The interviews were recorded, transcribed verbatim, and analyzed using qualitative content analysis to explore commonalities and differences in the experiences shared by the participants [42]. A critical friend procedure was used during the analysis procedure [43]. While the first author conducted the main analysis, the other authors asked questions during the process, promoting alternative interpretations. Finally, all authors discussed differences in coding, subcategories, and categories until consensus was reached.

### 2.7. Ethical Consideration

We certify that all applicable institutions and governmental regulations concerning the ethics of involving human volunteers were followed in the study. All participants were informed that participation was voluntary and gave informed written consent for use of the collected data. Ethical approval was obtained from The Ethics Committee at Umeå university (no. 2015/210-32, 2013-104-31M) for the Swedish participants. For the Norwegian participants, ethical approval was obtained from the Norwegian Centre for Research Data (16.04.2015/42831). This study was not preregistered.

## 3. Results

In the results section, we first provide descriptive statistics of the sample, the outcomes, and the antecedent conditions used in the fsQCA analyses. Secondly, we present results from the fsQCA analyses exploring necessary and/or sufficient conditions for the psychological, physical, and social benefits of the camp. Thirdly, we present results from the qualitative analysis. Lastly, we present results from the fsQCA analysis exploring the necessary and/or sufficient conditions for perceived fitness and exercise mastery six months after the camp.

### 3.1. Descriptive Statistics of Outcomes and Antecedent Conditions

Sample size, age, sex, and injury level are presented in Table 1 for the interviews, the pre/post survey, and the follow-up survey. The age range of the participants shows a large variation (from 15 years of age to 59).

The average scores of the outcomes immediately after the camp show that the participants reported considerably higher social benefits (SOB) from the camp than physical (PHY) and psychological (PSY) benefits. Table 2 shows the distribution of cases after calibration of the variables.

### 3.2. Physical, Psychological, and Social Benefits of the Camp

Table 3 shows which combination of conditions sufficiently explained the groups of participants who experienced physical, psychological, and social benefits of the camp. In the analysis, we included variables measuring age, year of injury, and the neurological level and degree of SCI to test whether we could observe group differences in the experienced social, physical, and psychological benefits of the camp. Based on previous research showing that people with paraplegia more often report being physically active and experience a greater degree of fitness [44], we expected group differences in terms of physical benefits of the camp, but not necessarily in social or psychological benefits. We also expected to observe differing experiences of benefits from the camp between newly injured participants and participants with longer experience with living with SCI.

Table 3 indicates the identified paths to experience benefits from the camp. All models had good model fit. The paths can be said to represent different groups of participants, although one participant can be placed in two groups. An example of this are participants in a1 and a3; the only thing that differs between the groups are n/a conditions, meaning that participants described by the conditions as portrayed in a3 could also be described as the conditions portrayed in a1. Most groups are categorized similarly in model (3a) and (3b) as portrayed by the paths (Table 3). The exception is in the social benefit model.

The number of paths, and the number of participants within the paths, indicates that the heterogeneity within the participant groups is also observable in the reported experiences from the camp. The group differences are better explained by describing the paths identified more in detail. The three first paths in models (3a) and (3b) represent participants with tetraplegic injury. Paths a1 and b1 indicate that most participants with tetraplegic injury experienced physical and psychological benefits from the camp independent of their age or years since injury if they were walking, cycling, using a wheelchair, or moving about in some other way at least 4 h per week and planning to become more physically active.

Paths a2 and b2 represent young participants (25 years or younger) with tetraplegic injury injured less than two and a half years ago (*n* = 2). For this group, their initial physical activity level was not relevant; however, they needed to at least plan for change to benefit physically and psychologically from the camp.

Paths a3 and b3 represent participants with tetraplegic injury above the age of 25 with more than two years since injury. Previous physical activity level seemed irrelevant for this group as both those physically active and not physically active experienced physical and psychological benefits from the camp. However, all participants within this group were at least planning to become more physically active prior to the camp.

Paths a4 and b5 represent participants with paraplegic injury. In both paths, the participants were 25 years old or older and injured relatively recently. For this group, it seemed important that they were not regularly physically active but in a stage of planning to become more active prior to the camp to experience physical and psychological benefits from the camp.

In model (3c), two paths to experience social benefits were identified. Path c1 represents the older participants (*n* = 11) independent of year since injury or injury level, and path c2 represents the participants injured two years or more prior to the camp independent of age or injury level. In both paths, the participants were physically active and at least at the contemplation stage of change prior to the camp.

Overall, the three models show the complexity of how conditions interact differently for different groups of participants. The condition that was most consistent throughout the paths was the participants’ initial stage of change. In four out of the five pathways, initial stages of change seemed to play an important role. The analysis of the interviews during the camp and qualitative responses in the surveys may illuminate the results further.

### 3.3. Content Analysis of Participants’ Experience of the Camp

**Figure 1:**
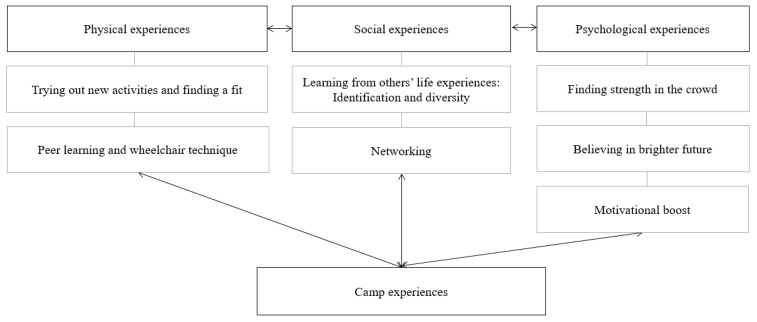
Camp experiences as described in the interviews with three categories and subcategories.

Based on content analysis [42] of the qualitative interviews, three overall categories emerged in the data: (1) physical experiences, (trying out new activities, peer learning, and wheelchair technique), (2) social benefits (learning from others’ life experiences; identification and diversity; networking), and (3) psychological benefits (finding strength in the crowd, believing in a brighter future, and motivational boost). The categories and subcategories are visualized in Figure 1.

#### 3.3.1. Physical Experiences

Trying out new activities and finding a fit: For most participants, the camp was an opportunity to test new activities that would otherwise be hard to come by: “There are many activities and there are many I have liked. It’s all about selecting something, trying it out to see if the activity is something for you. And sometimes you find one that fits” (Participant #7). For some of the participants that had not been as active prior to camp, the camp also involved increased physical exertion based on, among other factors, the many transfers between the various daily activities. As one participant elaborated: “You are much more physically active while you are here than what you are at home. This is important for me who is relatively newly injured” (Participant #2).

Peer learning and wheelchair technique: Most of the participants in the interviews, independent of years since injury and injury level, emphasized wheelchair techniques as an important learning outcome of the camp. Novice participants benefited from learning from the more experienced, and the more experienced participants benefited from teaching others and by learning new adjustments in techniques by observing other experienced participants. One of the participants, who was newly injured, explained:

“The physical therapist taught me different things, but it’s kind of-she’s walking. You learn more from those in wheelchairs, by observing how they do the techniques, up off the floor and in and out of the car and bed. Rocks and slopes, drive up and down... Rolling in their footsteps in a way” (Participant #3).

Even experienced wheelchair users had learning opportunities at the camp:

“I have (transferred into cars) for many years, and I had a car with me. People could train on transfers in my car if they wanted to and I could show them how I do it. Another participant also had experiences with transfers in and out of cars. This allowed me to compare and evaluate my technique and make smaller adjustment that I hadn’t thought about but that makes things a bit easier” (Participant #4).

Another participant, although experienced in being physically active in a wheelchair, also shared the experience of learning new skills at the camp: “It’s been a lot of fun. I didn’t think I would be able to drive down stairs, I hadn’t expected that. But now I do” (Participant #8).

#### 3.3.2. Social Benefits

Learning from others’ life experiences: Identification and diversity. Participants described peer learning of life skills throughout the camp. They learned by observing and talking to mentors and other participants with similar yet different life experiences: “The mentors have been great. There are different people with various disabilities so there is always someone that you can relate to—that is great” (Participant #7). We also wanted to understand how the participants experienced the age difference within the group. They all emphasized the age difference as a strength. As elaborated by one of the younger participants:

“The age difference is unimportant if you connect. People with similar interest, similar personalities—then age is no obstacle. Older people often have more experience, so it’s just good to meet people from different age groups, if you ask me” (Participant #1).

While some life skills sessions were organized, most life skills learning was based on peer learning outside of the organized activities. One participant elaborated on the social aspects of the camp going on in the background:

“What works or not if you have troubles. Nobody really teaches you. What you eat, how much and how late. If you stomach is bloated. It is hard to talk to your doctor about these things. However, when you talk to the woman in the same situation as you… Just to be able to talk to her you know. These are the conversations that goes on in the background” (Participant #4).

One of the participants that initially was hesitant to attend the camp explained that the social aspects of the camp were important: “I’m more positive to the camp now after spending some days here. The people have lot to say. As you get to know people better, they open up more. You talk more. That is very important” (Participant #1).

Networking: Several of the participants elaborated on how other participants and mentors at the camp served as motivators for new physical activities and sport opportunities available for them. Some participants found other participants with similar sport interests and several of the participants had made plans to meet at other sporting events.

#### 3.3.3. Psychological Benefits

Finding strength in the crowd: Several participants emphasized the psychological benefits of being in a larger group with other wheelchair users. This quote comes from a participant who previously hesitated to go to the local swimming pool because he thought it would draw attention to himself:

“Here, we are many together, everyone in a wheelchair, so you are just one of many. It is much easier to do things when we are together, and if you do it a couple of times, maybe it gets easier to do it when you get back home” (Participant #4).

One of the younger participants also emphasized the importance of being around other youths using a wheelchair:

“Where I live, it’s almost only me who is in a wheelchair. So, it is good to get to a place where there are more young people who are in a wheelchair. And get to know them and hear how they got injured and how they cope with it” (Participant #3).

Believing in a brighter future: One of the younger and newly injured participants emphasized that the greatest benefit from the camp was social and psychological. When asked to elaborate on the psychological benefits, he said: “In terms of believing in the future—it sort of opened up more—a more positive outlook… to look forward to a future, that it will be good. And that type of thinking” (Participant #1). The participant mentioned the social gatherings where participants shared everyday life experiences as particularly important: “I liked seeing that people are doing well. That they have taken higher education. That they have a girlfriend and things like that” (Participant #1). However, for some of the newly injured participants, being in a disability crowd was strange at first.

“I am new to this [read newly injured] so being in large crowds are hard for me. I try to get over it, so I’m doing better. In the beginning of the camp, I was a bit restrained. I have a good social life at home, so I thought: do I really need this? But then it is so much to learn and so nice to get to know new people that it disappears” (Participant #5)

Motivational boost: In the interviews, several participants emphasized the camp as an arena that boosted their motivation for being physically active:

“The good thing about such a camp like this is that you get a little, you get started again then. I’ve been training a bit on and off, but here you get a head start again in a way. And you get motivated to continue when you return home after such a stay” (Participant #2).

For the relatively newly injured (i.e., two years or less), several were searching for new ways of moving, being physically active, and being a competent wheelchair user in activities. The participants with longer experience in using a wheelchair can be divided into two groups. The first group of participants had adjusted well to life with SCI, but for several reasons struggled with being physically active in everyday life. The second group, the participants who exercised regularly in their local community alone or with others in a sport club, saw the camp as an opportunity to try out alternative activities that they might seek out when they returned home to their local communities.

### 3.4. Exercise Mastery and Fitness Six Months after the Camp

Table 4, model (4a) indicates six paths to the participants’ perceived exercise mastery six months after the camp. Common for all paths are the presence of a certain degree of physical activity and at minimum in a preparation stage of change. In three of the paths covering most of the participants (i.e., d1, d3, d4), all participants had experienced psychological and social benefits from the camp, and participants covered in the paths d1 and d4 also experienced physical benefits from the camp. However, the presence of the paths d2, d5, and d6 show that social and psychological benefit combined with a minimum of preparation stage and a certain degree of physical activity to be sufficient conditions, but not a necessary condition for experiencing mastery of exercise six months after the camp.

Model (4b) in Table 4 indicates four paths to perceived fitness with only six participants represented—all with paraplegic injury and all were physically active, and at a minimum of the preparation stage.

In both models, there are large variations in the relationships between the conditions describing the participant’s benefits from the camp and exercise mastery and perceived fitness six months later. The most consistent condition was the participants’ experience of social benefits from the camp. Only one participant reported experiencing mastery and perceived fitness without having experienced social benefits from the camp. Looking at the participants’ qualitative responses in the follow-up survey may illuminate the results as presented in the models (4a) and (4b). Based on the open-ended responses in the follow-up survey, most of the participants reported being in contact with fellow participants via Facebook. Some participants also met up at sport events and other physical activity camps. Thus, several of the participants actively used the social networks established at the camp six months later.

Finding a fit in activity does not mean that the participants find ways of engaging in the activity in their local community. Three of the six respondents that were not able to continue with the physical activity as intended post camp reported a lack of available sporting opportunities in their local community as an inhibiting factor.

## 4. Discussion

The current study aimed to describe the participants’ experiences of a Nordic physical AR camp. The camp reached a heterogenic group of participants (e.g., different age, gender, life histories, age at accident, time using a wheelchair, and expectations). This study demonstrated the complexity of experiences from such a camp.

### 4.1. Social and Psychological Benefits: Vicarious Experiences and Peer Mentoring

The main characteristics of the participants’ experiences from the AR camp, revealed from the combination of quantitative and qualitative data, were that most benefits were gained in the social and psychological area, with the social benefits yielding the highest scores. The qualitative data demonstrated a variety of social benefits that confirmed several of the tenets of Bandura’s social cognitive theory, such as learning from role models and vicarious experience [21]. In particular, those with recent injuries reported developing more comfort with their new identity and a more positive outlook on the future from meeting other individuals in a similar situation. Mentors and other participants also used gentle social persuasions, as well as acting to reduce stress and fearing stress reactions. All of this is directly in line with the advice of Bandura [21] to strengthen self-efficacy and is supported in a study demonstrating improvement of self-efficacy in persons with SCIs after a therapeutic outdoor recreation program [17].

Further, the use of peer mentors with more experience and skills, and therefore also likely with higher self-efficacy related to the activities introduced during the camp, is in line with research results on collective self-efficacy, which is a shared belief that individuals hold about the group they are in. According to Bandura [45], perceived collective efficacy in groups strengthens motivational commitment to their group’s missions, increases resilience to adversity, and yields results in performance accomplishments. Studies in areas such as computer learning and the academic field have demonstrated that grouping students with high self-efficacy and low self-efficacy together have positive effects on the group due to the high-efficacy individuals having modelling effects on other group members, and they are also more likely to transmit their efficacy beliefs through interactions with others [46]. Similar results have also been found in research on peer support for people with diabetes 2 [47], and within sports teams [48].

The results reflected that the participants with the most benefits were more likely to have a tetraplegic injury, but it was independent of their age or years since injury if they were at least at the preparation stage regarding readiness for physical activity.

In showing the various pathways to the outcomes, the analyses demonstrate the complexity of how conditions work differently for different groups of participants. The condition most consistent throughout the paths were participants’ initial stage of change. In four out of the five pathways to the outcomes, initial stages of change seemed to play an important role. The participation in this camp was voluntary, which most likely led to a selection of individuals with some degree of motivation or readiness for physical activity. According to the transtheoretical framework, different motivational approaches should be used at the various stages of readiness for physical activity to maximize motivation [20]. As all the participants initially were at least in the preparation stage, it is possible to use both cognitive processes (such as increasing awareness and knowledge) and behavioral processes (such as finding action possibilities, help decisions, receiving support, and overcome barriers) to increase the physical activity behavior. The qualitative data describe well how these processes were activated during the camp.

The social benefits seemed to be the most important, and the qualitative data gave ample examples of the importance of being together with and learning from others in a similar situation. It seems to create a feeling of being understood, and it makes it easier to open up about more sensitive issues. This supports the vicarious learning aspect of social cognitive theory [21], but it is not limited to learning from peer mentors. A lot of learning occurred in less formal ways. Age differences were perceived more as an asset than a problem because of exchange of experience.

### 4.2. Physical Benefits

There seemed to be more variation in the experience of physical benefits. It is natural that participants with more experience in being physically active with their injury experience less new learning than those that are younger, more recently injured, and with a higher-level injury (tetraplegic). However, the qualitative data demonstrated that even some of those with a lot of movement experience after injury learned new adjustments to their wheelchair techniques.

Taking part in the camp was a door-opener to new activities for many participants. Many physical activities are often quite expensive because of the adapted equipment needed. The opportunity to gain experiences in several activities helped the participants to find an activity that fitted their interests and functioning. Thus, AR camps serve as an important place to try out different activities without buying or applying for funding for expensive equipment before you know if the sport or activity is something for you. The findings from this study might contribute to better understanding how variations within the group of participants might result in different needs at the camp. This knowledge might help the organizer to better tailor the activities to the participant groups.

### 4.3. Is One Camp Enough to Change Perceived Exercise Mastery and Fitness?

Being somewhat physically active and at minimum in a preparation stage of change was a necessary and sufficient condition for experiencing exercise mastery and fitness six months after the camp. Interestingly, having a paraplegic injury was a necessary and sufficient condition for experiencing perceived fitness six months later. One reason may be that it is more difficult for individuals with tetraplegia to train fitness, e.g., normative data for physical capacity have been demonstrated to be lower for persons with tetraplegia than for those with paraplegia [49]. On the other hand, both participants with tetraplegia and paraplegia experienced exercise mastery six months later. Furthermore, there were large variations in the relations between the conditions describing the participants’ benefits from the camp and exercise mastery and perceived fitness six months later, with social benefit being the most consistent condition.

As indicated by this study, the outcomes of AR camp are complex and hard to measure. Finding ways to be physically active and become a confident wheelchair user (the concept of being en-wheeled is suggested [50]), takes time. The qualitative responses of the participants also indicated that there is a great variety of possibilities in different local environments, both in terms of the opportunities for organized para-sport and the social network for being active with other wheelchair users. For AR camps to be more than a boost and a door-opener for new activities, better follow up of participants may be needed when they get back to their local communities. In the Norwegian context, a way forward could be a cooperation with the Norwegian Olympic and Paralympic Committee and Confederation of Sports (NIF). Although the NIF strives to present opportunities in para-sport for everyone, as stated in their strategic plans, it is difficult to find local sports clubs that have a good enough offer [51]. The qualitative data in our study revealed a discrepancy between the NIF’s strategic plan and the reality the participants experienced depending on where they lived.

### 4.4. Strengths and Limitations of the Study

One strength of this study is the type of analysis used because it catches the complexity of the situation and illustrates the many ways participants may experience such a camp, and how many ways there are to achieve results. This demonstrates the need for other research methods than those that are based on average measures that tend to cover up such complexities. We need this type of information to tailor interventions to individual needs. We also consider the use of mixed methods a strength because the qualitative data add more nuances and personal meaning to the quantitative data. Combining quantitative and qualitative techniques within the same research design enables using the strengths of both methodologies [52].

A limitation of this study is the fact that the voluntary participation in the camp represents a selected group with a certain level of motivation for physical activity, something that limits generalization of the results. Another limitation is the lack of a control group, and that this type of approach does not rigorously measure change in physical activity level and motivation for physical activity after six months.

The relatively small number of participants could be seen as another limitation. However, both the fsQCA analyses and the additional qualitative data make that less of an issue in our opinion. On the other hand, some of the qualitative data were not collected under the most ideal circumstances. Some of them were taken from the open-ended part of a questionnaire which limits the possibilities of going in-depth with the responses, and some of the interviews were conducted under slight time pressure because they happened during breaks in a busy camp program.

The exact year of the data collection has not been presented to ensure the anonymity of the participants. It is a limitation that the data are somewhat old. However, they are more than 5 and less than 10 years old, and the basic structure of AR camps is still the same. Also, due to the limited research of this kind, we find it important to share the data that exist.

## 5. Conclusions

The experiences from the AR camp demonstrated complexity and several ways of benefiting depending on the individual situations of the participants. There are several ways to reach the goal, but some things also seem to be of a common nature, such as being motivated to change as a starting point. Social benefits were reported as the most common and with the highest scores and were unrelated to age and type and level of injury. Psychological benefits were quite close but seemed to be larger for those with tetraplegia. There was more variation in the physical benefits, which seemed to be related to amount of experience with physical activity with the SCI, but also some of the more experienced could learn something new, such as adjustment of wheelchair technique. To perceive exercise mastery or fitness six months after the camp, it seemed that at least being in a preparation stage of change and having a moderate level of physical activity to be both necessary and sufficient conditions. Perceived fitness was only experienced by those with injuries at a paraplegic level, but both persons with paraplegia and tetraplegia injury experienced exercise mastery.

## Figures and Tables

**Table 1:** Characteristics of participants.

	Interviews	Pre-/Post-Survey	Follow-Up Survey
Sample size	8	23	18
Age: Min–Max [Mean/St.D.]	15–25 [21.13/7.41]	15–59 [30.46/12.52]	15–59 [31.00/13.89]
Sex: Number [Percentage] Men	7 [87.5]	14 [58.3]	11 [61.1]
Injury level: Number [Percentage]			
Paraplegic incomplete	1 [12.5]	7 [29.2]	5 [27.8]
Paraplegic complete	4 [50.0]	8 [33.3]	6 [33.3]
Tetraplegic incomplete		2 [8.3]	2 [11.1]
Tetraplegic complete	3 [37.5]	7 [29.2]	5 [27.8]

**Table 2:** Descriptive statistics of the outcomes and the pre- and post-camp conditions.

Conditions	Code	Mean/SD	Minimum	Maximum	LC/CT/UT
Outcomes After Camp					
psychological benefits	PSY	6.03/3.05	0.00	10.00	0/5.01/10
physical benefits	PHY	6.51/2.88	0.00	10.00	0/5.01/10
social benefits	SOB	8.53/1.72	3.60	10.00	0/5.01/10
Pre-camp variables					
age	AGE	30.46/12.52	15.00	59.00	15/25.5/59
years since injury	YIN	7.17/9.51	0.50	40.00	0/2.1/40
injury level	INJ	2.63/1.20	1.00	4.00	1/2.5/4
physical activity	PAC	2.46/1.02	1.00	4.00	1/1.5/4
stages of change	SOC	4.46/0.978	2.00	5.00	1/2.5/4
Six-month Follow-up Outcome					
exercise mastery	MAS	3.05/0.61	1.60	4.00	1/2.5/4
perceived fitness	FIT	2.48/0.73	1.33	4.00	1/2.5/4
Post-camp variables					
age	AGE2	31.00/13.89	15.00	59.00	15/25.5/59
years since injury	YIN2	4.53/4.37	1.00	14.00	0/2.1/40
injury level	INJ2	2.61/1.95	1.00	4.00	1/2.5/4
physical activity	PAC2	2.72/0.89	1.00	4.00	1/1.5/4
stages of change	SOC2	4.50/0.79	3.00	5.00	1/2.5/4

Note. LT = lower threshold; CT = crossover threshold; UT = upper threshold.

**Table 3:** Sufficient paths for experiencing physical, psychological, and social benefits of the camp.

		(3a) Physical Benefits					(3b) Psychological Benefits					(3c) Social Benefits	
Pre-camp variables	AGE	n/r	◊	♦	◊	♦	n/r	◊	♦	◊	♦	♦	n/r
	YIN	n/r	◊	♦	♦	◊	n/r	◊	♦	♦	◊	n/r	♦
	INJ	◊	◊	◊	♦	♦	◊	◊	◊	♦	♦	n/r	n/r
	PA	♦	n/r	n/r	◊	◊	♦	n/r	n/r	◊	◊	♦	♦
	SOC	♦	◊	♦	♦	♦	♦	◊	♦	♦	♦	♦	♦
Model fit	Incls.	0.973	1.000	0.982	0.939	0.984	0.897	0.998	0.959	0.964	0.979	1.000	1.000
	PRI	0.953	1.000	0.962	0.737	0.961	0.804	0.996	0.910	0.858	0.947	1.000	1.000
	Cov.r	0.425	0.135	0.312	0.197	0.238	0.426	0.147	0.331	0.220	0.257	0.472	0.461
	Cov.u	0.132	0.048	0.016	0.012	0.038	0.115	0.052	0.018	0.020	0.042	0.033	0.022
	*n*	6	2	3	1	1	6	2	3	1	1	11	10
	Paths	a1	a2	a3	a4	a5	b1	b2	b3	b4	b5	c1	c2

Note. Physical benefits model (3a): Incls = 0.956, PRI = 0.928, Covs = 0.621. Psychological benefits model (3b): Incls = 0.913, PRI = 0.854, Covs = 0.644. Social benefits model (3c): Incls = 1.000, PRI = 1.000, Covs = 0.748. ♦ = above the threshold, ◊ = below the threshold, n/r = not relevant. YIN = years since injury, INJ = injury level, PA = physical activity, SOC = stage of change.

**Table 4:** Sufficient paths to experiencing exercise mastery and perceived fitness six months after the camp.

		(4a) Exercise Mastery						(4b) Perceived Fitness			
Post-camp variables	PHY	♦	◊	n/r	♦	♦	◊	◊	n/r	◊	♦
	PSY	♦	n/r	♦	♦	◊	◊	n/r	♦	◊	◊
	SOB	♦	♦	♦	♦	◊	♦	♦	♦	♦	◊
	AGE	♦	◊	♦	n/r	◊	♦	◊	◊	♦	◊
	YIN	n/r	♦	♦	◊	◊	◊	♦	♦	◊	◊
	INJ	n/r	♦	◊	◊	♦	♦	♦	♦	♦	♦
	PA2	♦	♦	♦	♦	♦	♦	♦	♦	♦	♦
	SOC2	♦	♦	♦	♦	♦	♦	♦	♦	♦	♦
Model fit	Incls	0.840	1.000	0.952	0.974	1.000	1.000	0.983	0.913	0.922	0.987
	PRI	0.702	1.000	0.810	0.920	1.000	1.000	0.936	0.538	0.732	0.941
	Cov.r	0.489	0.231	0.295	0.415	0.159	0.269	0.342	0.336	0.340	0.223
	Cov.u	0.083	0.058	0.007	0.109	0.031	0.085	0.058	0.042	0.112	0.43
	*n*	7	2	3	4	1	2	2	2	2	1
	Paths	d1	d2	d3	d4	d5	d6	e1	e2	e3	e4

Notes. Exercise mastery model (4a): incls = 0.893, PRI = 0.831, Covs = 0.785. Perceived fitness model (4b): incls = 0.900, PRI = 0.769, Covs = 0.528. ♦ = above the threshold, ◊ = below the threshold, n/r = not relevant. PHY = physical benefits, PSY = psychological benefits, SOB = social benefits, AGE = age, YIN = years since injury, INJ = injury level, PA2 = physical activity sixth months after the camp, SOC2 = stage of change six months after the camp.

## Data Availability

Data are unavailable due to privacy restrictions.
